# Benchmarking peak calling methods for CUT&RUN

**DOI:** 10.1093/bioinformatics/btaf375

**Published:** 2025-06-26

**Authors:** Amin Nooranikhojasteh, Ghazaleh Tavallaee, Elias Orouji

**Affiliations:** Princess Margaret Cancer Centre, University Health Network (UHN), Toronto, ON M5G 1L7, Canada; Princess Margaret Cancer Centre, University Health Network (UHN), Toronto, ON M5G 1L7, Canada; Princess Margaret Cancer Centre, University Health Network (UHN), Toronto, ON M5G 1L7, Canada

## Abstract

**Motivation:**

Cleavage Under Targets and Release Using Nuclease (CUT&RUN) has rapidly gained prominence as an effective approach for mapping protein-DNA interactions, especially histone modifications, offering substantial improvements over conventional chromatin immunoprecipitation sequencing (ChIP-seq). However, the effectiveness of this technique is contingent upon accurate peak identification, necessitating the use of optimal peak calling methods tailored to the unique characteristics of CUT&RUN data.

**Results:**

Here, we benchmark four prominent peak calling tools, MACS2, SEACR, GoPeaks, and LanceOtron, evaluating their performance in identifying peaks from CUT&RUN datasets. Our analysis utilizes in-house data of three histone marks (H3K4me3, H3K27ac, and H3K27me3) from mouse brain tissue, as well as samples from the 4D Nucleome database. We systematically assess these tools based on parameters such as the number of peaks called, peak length distribution, signal enrichment, and reproducibility across biological replicates. Our findings reveal substantial variability in peak calling efficacy, with each method demonstrating distinct strengths in sensitivity, precision, and applicability depending on the histone mark in question. These insights provide a comprehensive evaluation that will assist in selecting the most suitable peak caller for high-confidence identification of regions of interest in CUT&RUN experiments, ultimately enhancing the study of chromatin dynamics and transcriptional regulation.

**Availability and implementation:**

The CUT&RUN data generated in this study have been deposited in the Gene Expression Omnibus (GEO) under the accession number GSE282809. All the 4D Nucleome datasets can be obtained from the 4D Nucleome Data Portal (https://data.4dnucleome.org/). All scripts used for data processing, figure generation, and analysis are available in the following GitHub repository: https://github.com/OroujiLab/CUTandRun_Peak_Calling/, and have also been archived on Zenodo.

## 1 Introduction

In recent years, Cleavage Under Targets and Release Using Nuclease (CUT&RUN) has emerged as a powerful alternative to traditional ChIP-seq for profiling protein-DNA interactions, particularly histone modifications (Meers *et al.* 2019). This technique offers a higher signal-to-noise ratio (SNR), requires less input material, and provides a more precise mapping of protein-DNA binding sites (Skene *et al.* 2018). However, as with any genomic technique, the accuracy and efficiency of data analysis heavily depend on the tools used to call peaks from the raw sequencing data. Proper peak calling is crucial for identifying regions of interest, such as transcription factor binding sites or histone modifications, but choosing the best peak calling method for a given dataset remains a challenge.

The diversity of peak calling algorithms and their underlying assumptions can lead to significantly different results when applied to the same dataset. For instance, traditional ChIP-seq peak callers like MACS2 are widely used but may not be fully optimized for CUT&RUN’s unique signal characteristics (Zhang *et al.* 2008). Conversely, newer tools such as SEACR, which was specifically designed for CUT&RUN data, claim to better exploit the sparse but strong signal profile typical of this technique (Meers *et al.* 2019). Other tools, like GoPeaks and LanceOtron, bring their own strengths, such as robust binomial modeling or machine learning-based predictions, further complicating the decision of which tool is best suited for analyzing CUT&RUN datasets (Hentges *et al.* 2022, Yashar *et al.* 2022). These tools employ distinct computational strategies to identify protein-binding sites: MACS2 employs Poisson distribution modeling to identify significant genomic regions by comparing enriched regions against local background with IgG controls, evaluating read density distributions to determine peak significance. SEACR implements empirical thresholding with IgG inputs, establishing statistical cutoffs to discriminate true binding events from background noise. GoPeaks applies machine learning patterns, utilizing trained models to recognize characteristic binding patterns, while LanceOtron leverages deep neural networks to identify peaks without requiring control samples, both tools employing computational learning approaches to distinguish genuine binding sites from background signals.

Given these diverse approaches, a systematic comparison is needed to evaluate their effectiveness. Therefore, the aim of this study is to benchmark four peak calling methods, MACS2, SEACR, GoPeaks, and LanceOtron on their ability to accurately and efficiently detect peaks from CUT&RUN data. We focus on CUT&RUN experiments profiling three histone marks, H3K4me3, H3K27ac, and H3K27me3, using two replicates per mark from mouse brain tissue that are generated in-house along with four samples with different number of replicates from the 4D Nucleome database (a total of 15 samples across the three histone marks) (Dekker *et al.* 2017). By comparing the number of peaks called, peak length, signal enrichment, and reproducibility across the methods, we aim to determine which tool is the most effective for CUT&RUN data analysis. Our findings will provide insights on parameters to consider while selecting peak calling tools and help improve the reliability of CUT&RUN experiments in studying chromatin dynamics and transcriptional regulation.

In this article, we address the following key questions: (i) Which peak calling method identifies the highest number of biologically relevant peaks? (ii) How consistent are these peaks across replicates? (iii) What are the trade-offs between peak sensitivity and specificity across different tools? By systematically comparing these tools on the same dataset, we seek to provide insights into which peak calling method is best suited for high-confidence peak identification in CUT&RUN experiments.

## 2 Materials and methods

### 2.1 Sample collection and experimental design

#### 2.1.1 Mouse samples

Adult C57BL6 mice were used for this study. Fresh brain tissue was obtained from female mice of 8–10 weeks of age. All animal samples obtained in this study complied the relevant ethical regulations approved by the institutional ethics committee and Research Ethics Board at the University Health Network (UHN). The chromatin profiling in this study targeted three specific histone modifications: H3K4me3; associated with active transcription start sites and promoters, H3K27ac; a marker for active enhancers and promoters and H3K27me3; related to repressive chromatin domains and silenced genes. Two biological replicates were prepared for each histone mark. These histone marks were assayed using anti-H3K4me3 (Abcam, Cat. ab8580), anti-H3K27ac (Abcam, Cat. ab4729) and anti-H3K27me3 (Diagenode, Cat. C15410069). The most commonly used and optimized CUT&RUN protocol (Meers *et al.* 2019) was used for all samples, ensuring minimal background noise and high SNR typical of CUT&RUN experiments.

#### 2.1.2 4D Nucleome datasets

For this study, we obtained publicly available CUT&RUN datasets from the 4D Nucleome Data Portal (https://data.4dnucleome.org/). We selected data for four specific samples, each with technical replicates, for which data was available for all three histone marks investigated in this study: H3K4me3, H3K27ac, and H3K27me3. The files were retrieved by accessing the unique dataset identifiers listed in the 4D Nucleome database. Each dataset was then systematically downloaded using the portal’s API, ensuring data integrity through verification of checksum values. The BAM files were subsequently processed for quality control and peak calling to facilitate further analysis and integration in our research. All data processing was conducted on UHN’s high-performance computing infrastructure, ensuring reproducibility and consistency across the analyses. Table S1, available as supplementary data at *Bioinformatics* online shows details of the samples used in this study.

#### 2.1.3 Library complexity and IgG normalization

To provide insight into sequencing depth and enrichment specificity, we assessed the total number of reads per library and calculated IgG enrichment ratios across all datasets. As summarized in Table S2, available as supplementary data at *Bioinformatics* online, IgG-normalized signal-to-background ratios were computed to assess assay quality and specificity. Table S3, available as supplementary data at *Bioinformatics* online lists the total number of reads obtained for each CUT&RUN library, spanning both the in-house and 4D Nucleome datasets. These metrics were used to evaluate library complexity and inform downstream filtering and normalization decisions.

### 2.2 CUT&RUN protocol

The CUT&RUN procedure was performed following the standard protocol described by Meers *et al.* (2019). Fresh brain tissue was homogenized, and nuclei were isolated using a lysis buffer optimized for brain tissue, followed by incubation with Concanavalin A-coated magnetic beads. The isolated nuclei were then incubated overnight at 4°C with the respective antibodies targeting the histone marks H3K4me3, H3K27ac, H3K27me3, and IgG. Antibody concentrations were optimized according to the manufacturer’s recommendations. Following antibody binding, the nuclei were treated with Protein A/G-MNase fusion protein to cleave the chromatin at the antibody-bound sites. After MNase activation, the DNA fragments were released and collected for downstream sequencing.

### 2.3 Sequencing and data processing

#### 2.3.1 Library preparation and sequencing

CUT&RUN libraries were prepared from the released DNA fragments using the NEBNext Ultra II DNA Library Prep Kit (New England BioLabs). The libraries were sequenced using Illumina NovaSeq 6000 with paired-end 50 base pair (50-PE) reads at a sequencing depth of ∼40 million reads per sample.

#### 2.3.2 Data preprocessing

Raw sequencing reads were processed using the nf-core/cutandrun pipeline (v3.2.2) with default settings (Meers *et al.* 2019). FASTQ files were quality checked using FastQC (v0.11.9), followed by adapter trimming using Trim Galore (v0.6.10). Reads were then aligned to either the mm10 mouse or hg38 human reference genome using Bowtie2 (v2.4.1) with default parameters, including the use of both target and spike-in genomes. The resulting BAM files were sorted and indexed with SAMtools (v1.11), and duplicate reads were marked using Picard (v3.1.1).

### 2.4 Peak calling methods

Four peak calling methods were benchmarked in this study: MACS2, SEACR, GoPeaks, and LanceOtron. Each method was run using default parameters unless otherwise specified, with the goal of determining which method most efficiently detects peaks in CUT&RUN datasets. This study included both mouse (mm10) and human (hg38) samples. Below, we describe the peak calling parameters used for each tool.

MACS2 (Model-based Analysis of ChIP-Seq) (MACS2 v2.2.9.1) was used for peak calling on BAM files aligned to both the mm10 and hg38 genomes. For each BAM file, peaks were called with the following parameters: paired-end BAM format (-f BAMPE), genome sizes set to 2.7e9 (mm10) and 3.1e9 (hg38), and a false discovery rate cutoff of 0.05 (-q 0.05). For H3K27me3-marked samples, the –broad option was applied to identify broad peaks, while no additional broad peak option was specified for other histone marks.

SEACR (Sparse Enrichment Analysis for CUT&RUN) (SEACR v1.3) was used to call peaks by first converting BAM files to bedGraph format with BEDTools genomecov (Quinlan and Hall 2010). SEACR was then run in stringent mode for both mm10 and hg38 alignments, with a false discovery rate threshold of 0.01, generating output files in the stringent peak format for each genome.

GoPeaks (GoPeaks v1.1) peak calling was performed on BAM files aligned to mm10 and hg38. BAM files were processed in batches, excluding index (.bai) files. For H3K27me3-marked samples, the –mdist 3000 and –broad options were applied to account for the broad distribution of this histone mark, while other histone marks used –mdist 1000. Peak files in BED format were generated and saved for each genome alignment, with an FDR threshold of 0.01.

LanceOtron (LanceOtron v1.2.7) used bigWig format as input. For each BAM file aligned to either mm10 or hg38, a bigWig file was generated using bamCoverage from Deeptools package, which was then used as input for LanceOtron to identify peaks (Ramírez *et al.* 2016). Peaks were called and saved in BED format, with bigWig files moved to a separate directory and BED files stored in the output directory. BigWig files were processed using bamCoverage, and peaks were loaded into R as GRanges objects (Lawrence *et al.* 2013). Peak files in either BED, narrowPeak, or broadPeak formats were imported for each tool. Signal extraction from the bigWig files was performed over the peak regions, and background noise was estimated using randomly selected regions without peaks.

The SNR was calculated by dividing the signal values by the mean background noise for each condition, and the results were saved for further analysis.

### 2.5 Signal-to-noise ratio calculation


$$\text{SNR}=\frac{\left(\frac{S_{\text{target}}}{S_{\lambda ,\text{target}}}\right)}{\left(\frac{S_{\text{IgG}}}{S_{\lambda ,\text{IgG}}}\right)}=\frac{S_{\text{target}}\times S_{\lambda ,\text{IgG}}}{S_{\text{IgG}}\times S_{\lambda ,\text{target}}}$$

where,

-
$S_{\text{target}}$ : Number of reads mapped to the genome (or target regions) in the target antibody sample.
-
$S_{\lambda ,\text{target}}$ : Number of reads mapped to the spike-in DNA in the target antibody sample.
-
$S_{\text{IgG}}$ : Number of reads mapped to the genome (or target regions) in the IgG control sample.
-
$S_{\lambda ,\text{IgG}}$ : Number of reads mapped to the spike-in DNA in the IgG control sample.

In the absence of spike-in controls, background signal was estimated using randomly selected genomic regions of matching size, generated with bedtools shuffle. This method accounts for local chromatin biases and was applied consistently across all datasets, including both in-house and 4D Nucleome samples. SNR values were log_2_-transformed for visualization to better reflect the dynamic range of enrichment.

### 2.6 Generation of consensus peak dataset as the ground truth

To evaluate the performance of each peak calling method, we constructed a consensus peak set to serve as a reference ground truth for benchmarking. Overlapping regions between peaks from different methods were identified based on any shared base pair. For generating the consensus peak set, we employed a more stringent approach where we first identified all genomic positions covered by peaks from any method, then calculated the number of methods that called a peak at each position. we converted all peak regions into genomic coverage by generating binary coverage vectors for each method, where each genomic position was marked as covered or uncovered. A genomic region was considered part of the consensus peak set if it was identified by at least three out of the four peak calling methods. To address fragmentation, closely spaced consensus regions were merged if separated by less than or equal to 50 bp. This approach aimed to define high-confidence regions consistently supported by multiple peak callers and minimizing false positive regions.

Next, we identified peaks from all possible combinations of three peak callers, generating four sets of consensus peaks. The union of these overlapping regions was defined as the final consensus peak set. We then evaluated each individual peak caller’s performance against this consensus using three metrics: precision (proportion of called peaks that overlap with consensus peaks), recall (proportion of consensus peaks detected by each caller), and F1 score (harmonic mean of precision and recall). The analysis was visualized using Venn diagrams to display the overlap between different peak callers and the summary of these metrics were plotted for each peak calling method across three different marks separately.

### 2.7 Precision, recall compositional normalization, and ternary plot visualization

The precision and recall scores derived from each peak-calling method were normalized to represent the relative contributions of three histone modifications (H3K27ac, H3K4me3, and H3K27me3) in a ternary plot. Specifically, let $p_{i,j}$ denote the precision score for histone mark i in replicate j, where i∈{H3K27ac, H3K4me3, H3K27me3}, and j represents the individual replicates for each condition. The normalized score $q_{i,j}$ for each histone mark i was computed as follows:


$$q_{i,j}=\frac{p_{i,j}}{\sum_{i=1}^{3}p_{i,j}},$$

where the denominator represents the sum of precision scores across the three histone marks for each replicate, ensuring that the normalized scores for each replicate sum to unity:


$$\sum_{i=1}^{3}q_{i,j}=1.$$

This compositional normalization method allows for visualization of the relative precision of the peak-calling methods across the three histone marks, highlighting their differential contributions. The resulting ternary plot provides a comprehensive overview of the performance of the peak-calling methods, enabling comparison across multiple conditions and replicates.

## 3 Results

### 3.1 Peak comparison and evaluation

#### 3.1.1 Metrics for benchmarking

The performance of each peak caller was evaluated based on the following criteria: (i) **Total Number of Peaks**: The total number of peaks called by each method was quantified to assess the sensitivity of each peak caller in identifying potential regions of interest across the dataset. (ii) **Peak Length Distribution**: The distribution of peak lengths was analyzed for each method to compare peak length patterns and identify differences in resolution and detection of broad versus narrow peaks across the genome. (iii) **SNR**: Enrichment of signal over background noise was evaluated by analyzing the read density across peaks. This metric helps in determining each method’s ability to distinguish true peaks from background noise, thus indicating overall signal clarity. (iv) **Precision, Recall, and F1 Score Metrics**: These metrics were calculated by comparing identified peaks against a reference set of high-confidence peaks. Precision measures the fraction of true positives among all called peaks, recall measures the fraction of true positives among actual peaks, and the F1 Score provides a balance between precision and recall. (v) **Overlap Analysis Between Methods**: Pairwise comparisons were conducted to assess the overlap in peak calls across different methods. This analysis provided insights into the consistency and specificity of each peak caller. (vi) **Overlap Analysis Between Replicates**: The reproducibility of peaks between biological replicates was assessed for each method using the Irreproducible Discovery Rate (IDR). IDR analysis determined the consistency of peak detection between replicates, which is crucial for assessing method reliability.

### 3.2 Total number of peaks called

The total number of peaks called by each peak caller, MACS2, SEACR, GoPeaks, and LanceOtron showed marked differences across the histone marks H3K4me3, H3K27ac, and H3K27me3. LanceOtron identified the highest number of peaks across all histone marks, with an average of 110 876 for H3K4me3, 367 023 for H3K27ac, and 281 545 for H3K27me3. This was consistent across both biological replicates for each histone mark, indicating LanceOtron’s sensitivity to the sparse, high-intensity signal typical of CUT&RUN datasets.

**Figure 1.**
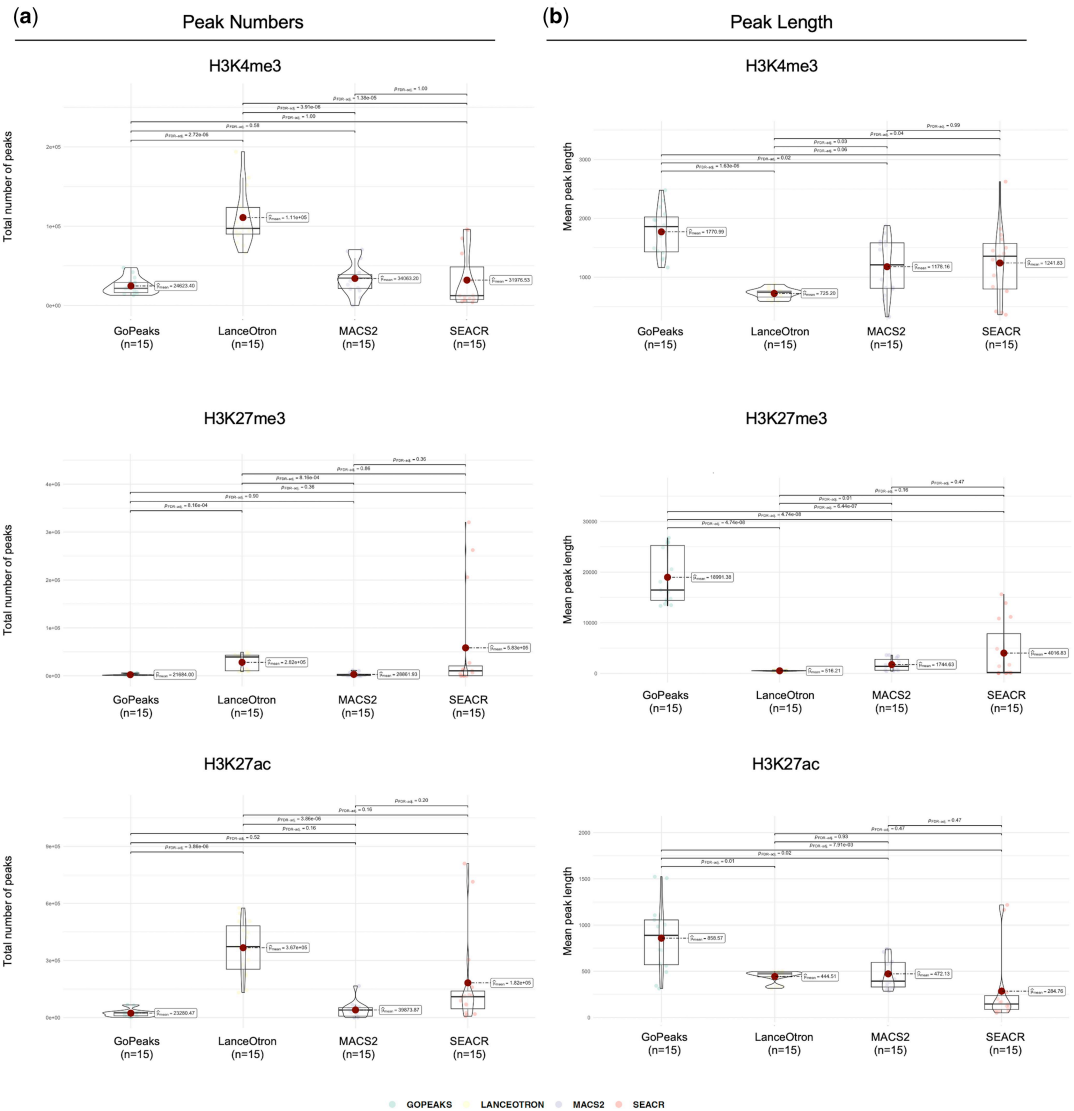
Distribution of (a) peak numbers and (b) peak lengths for three histone marks, analyzed using four peak-calling methods.

**Figure 2.**
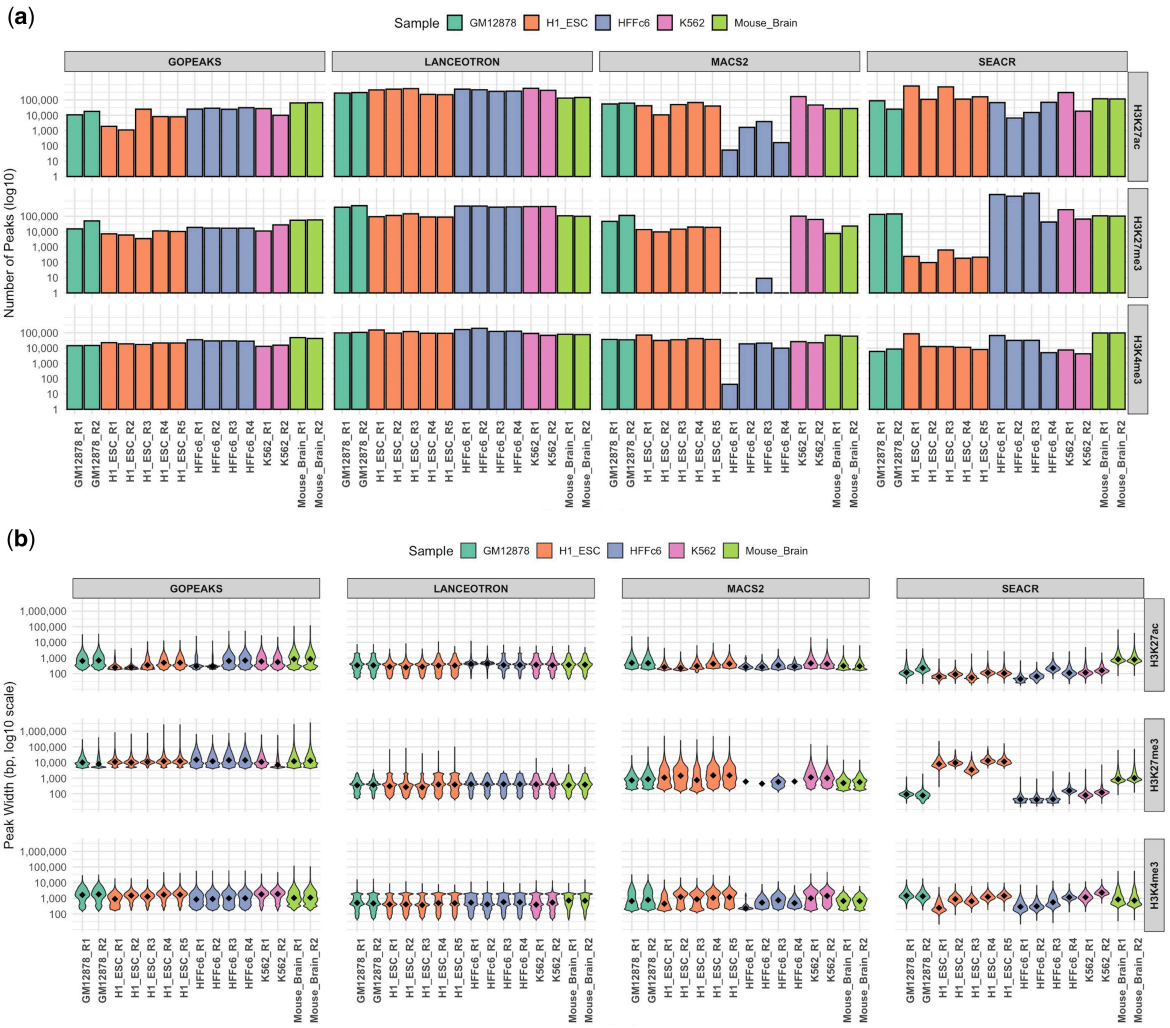
Distribution of (a) peak numbers and (b) peak lengths across all individual samples and their replicates, using all four peak-calling methods.

In contrast, GoPeaks called the fewest peaks, with significantly lower numbers across all histone marks: 24 623 for H3K4me3, 23 280 for H3K27ac, and 21 684 for H3K27me3. The peak counts from MACS2 and SEACR were intermediate, with MACS2 detecting 34 063 peaks on average for H3K4me3, 39 873 for H3K27ac, and 28 861 for H3K27me3, while SEACR identified 31 976, 182 261, and 583 414 peaks for the same marks, respectively. These trends suggest that GoPeaks is the most stringent in peak detection, potentially at the expense of missing biologically relevant but weaker peaks, whereas LanceOtron captures a broader spectrum of peaks, which may include more false positives **(**Figs 1a and 2a).

**Figure 3.**
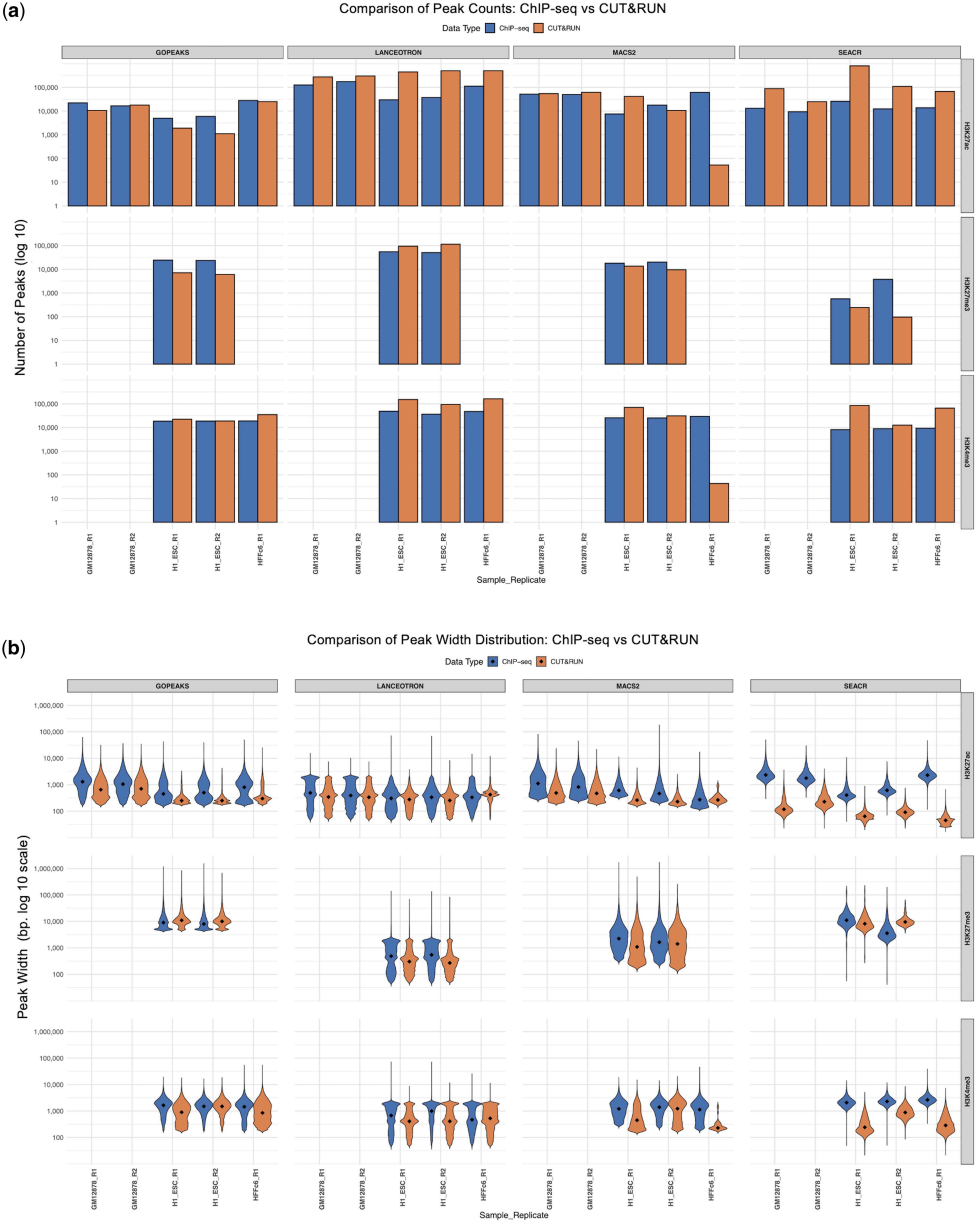
Comparison of CUT&RUN and ChIP-seq datasets in terms of (a) peak counts and (b) peak lengths, using data from the 4D Nucleome project.

To further contextualize these findings, we compared peak counts between CUT&RUN and matched ChIP-seq datasets from the 4D Nucleome project, where available. As shown in Fig. 3a, while trends in peak counts were generally consistent across methods for histone marks characterized by narrow peaks (H3K27ac and H3K4me3), notable discrepancies emerged for the broad histone mark H3K27me3. Specifically, LanceOtron consistently identified more peaks in CUT&RUN data compared to ChIP-seq, whereas SEACR showed substantially fewer peaks. These contrasting trends underscore that peak-calling outcomes are markedly influenced by both the choice of analytical method and the assay-specific signal properties inherent to CUT&RUN versus ChIP-seq techniques.

### 3.3 Peak length distribution

The distribution of peak lengths varied significantly between the peak callers. GoPeaks produced consistently longer peaks compared to MACS2, SEACR, and LanceOtron across all histone marks. The mean peak length for H3K4me3 using GoPeaks was 1770 bp, while LanceOtron produced much narrower peaks with a mean length of 725 bp. For H3K27ac, GoPeaks’s peak lengths were similarly broader, with a mean length of 858 bp compared to the narrowest peaks in SEACR with the mean of 284 bp. This trend continued with H3K27me3, where GoPeaks identified much broader repressive regions, averaging 18 991 bp in length, while LanceOtron called the narrowest peaks at 516 bp. With the exception of the H3K27ac mark, MACS2 and SEACR showed peak length distributions that were intermediate between GoPeaks and LanceOtron, with MACS2 detecting mean peak lengths of 1178 bp for H3K4me3, 472 bp for H3K27ac, and 1744 bp for H3K27me3, while SEACR detected 1241 bp, 284 bp, and 4016 bp for the respective marks **(**Figs 1b and 2b).

These observations are further illustrated in Fig. 3b, which compares peak length distributions between CUT&RUN and 4D Nucleome ChIP-seq datasets. In most cases, CUT&RUN peaks were sharper and narrower, reflecting the assay’s lower background and higher resolution. The consistency of peak length trends across data types supports the reliability of CUT&RUN for capturing histone modification profiles, while highlighting the importance of selecting an appropriate peak caller based on the biological characteristics of the histone mark under study.

To extend this analysis, we next evaluated how sensitive each peak caller is to user-defined input parameters, such as statistical thresholds, which can substantially influence peak detection outcomes. Adjusting the q-value threshold in MACS2 revealed that increasing the cutoff progressively increased the number of peaks identified, with a more pronounced effect observed for broad peak types. The narrow peak calls remained more stable, indicating MACS2's narrow peak detection is less sensitive to threshold variation, while broad peak detection is more affected by relaxed stringency **(**Fig. S1a, available as supplementary data at *Bioinformatics* online**).** We assessed GoPeaks performance by adjusting the *P*-value threshold from .001 to .1. As expected, relaxing the *P*-value threshold led to a modest increase in the total number of peaks called, particularly for broad peaks. However, the peak count remained relatively stable across the range, suggesting GoPeaks' default settings already strike a conservative balance between sensitivity and specificity (Fig. S1b, available as supplementary data at *Bioinformatics* online). We analyzed SEACR using its two operational modes (relaxed and stringent) and varied the threshold parameter from 0.005 to 0.05. SEACR displayed more dramatic shifts in peak counts between the relaxed and stringent modes than between different thresholds within the same mode. This highlights that SEACR’s mode setting has a stronger influence on peak detection than fine-tuning of the numeric threshold alone (Fig. S1c, available as supplementary data at *Bioinformatics* online).

### 3.4 Signal-to-noise ratio

**Figure 4.**
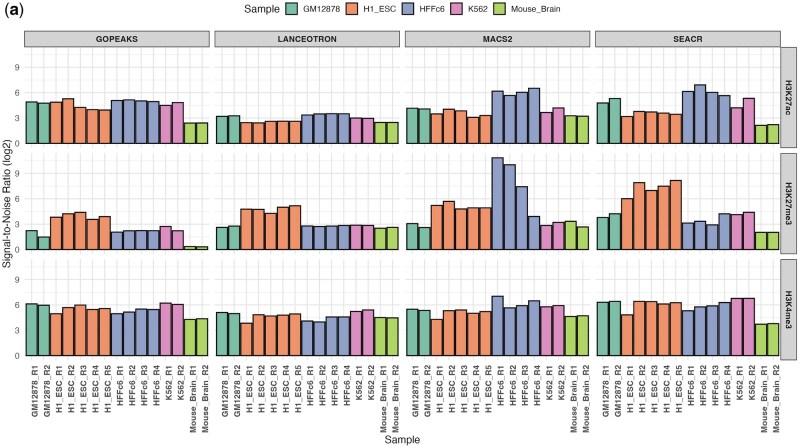
Signal to noise ratio (SNR) across all marks using four peak calling methods.

To evaluate the signal enrichment achieved by each peak caller, we quantified the SNR using bigWig signal tracks and peak data for H3K27ac, H3K4me3, and H3K27me3 across all biological replicates and peak calling methods (Fig. 4). SNR quantifies the contrast between signal intensity within peak regions and corresponding background regions, providing a direct measure of enrichment quality.

SNR analysis revealed distinct differences in sensitivity for detecting peaks across three histone marks (H3K27ac, H3K27me3, and H3K4me3). SEACR consistently achieved the highest SNR across all marks, underscoring its superior sensitivity and suitability for high-confidence peak detection, particularly in complex chromatin contexts involving both active and repressive marks.

MACS2 demonstrated high SNR for active histone marks (H3K27ac and H3K4me3), indicating strong signal enrichment for transcriptionally active regions. GoPeaks and LanceOtron provided moderate, stable SNR values across all marks, reflecting consistent performance but with lower sensitivity than SEACR, especially for marks with subtle signal patterns like H3K27me3.

### 3.5 Overlap analysis between methods

**Figure 5.**
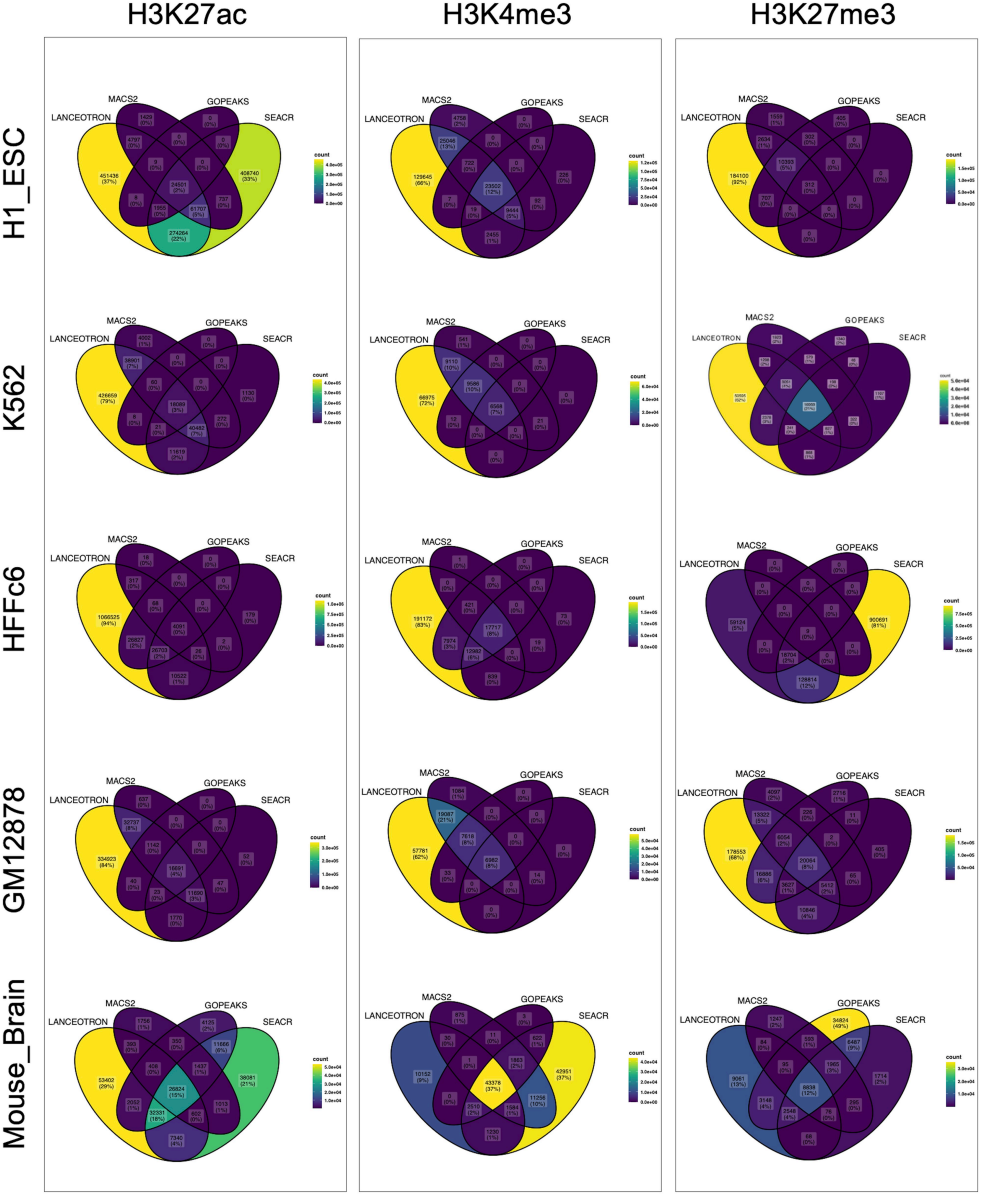
Venn diagrams showing peak overlaps among the four peak calling methods across samples. For each sample and histone mark, the union of peaks from all biological replicates was used to assess the overlap between methods.

Figure 5 presents Venn diagrams illustrating the overlap among the four peak calling methods for each sample. These diagrams, generated separately for each histone mark (H3K27ac, H3K4me3, and H3K27me3) and each sample (H1_ESC, K562, HFFc6, GM12878, and Mouse-Brain), provide key insights into the similarities and discrepancies in peak detection across the methods.

The Venn diagrams for H3K4me3 in the Mouse_Brain and H1_ESC samples show a considerable overlap among all four peak calling methods, with 43,378 (37%) and 23 502 (12%) peaks, respectively, shared among the four methods.

For H3K27me3, the highest percentage of overlapping peaks was observed in K562 with 16 959 (21%), followed by 8838 (12%) in Mouse_Brain and 20 064 (8%) in GM12878.

For H3K27ac, the percentage of shared peaks among all four methods did not exceed 4%, with the exception of the Mouse_Brain sample, which had 26 824 (15%) shared peaks.

A consistent pattern across histone marks and samples was observed, with GoPeaks detecting fewer peaks. Conversely, LanceOtron tended to call the highest number of peaks, generating the highest number of peaks in 12 out of 15 samples across all three histone marks.

The overlap analysis reveals that, despite the inherent differences among the peak callers, a substantial number of peaks are shared across 2–3 methods, particularly for the active histone marks H3K27ac and H3K4me3. The overlap for H3K27me3, a repressive mark, is notably lower, which might reflect the broader and more variable nature of these peaks **(**Fig. 5 and Table S4, available as supplementary data at *Bioinformatics* online).

### 3.6 Precision, recall, and F1 score metrics

Using the consensus peak set as the reference, we assessed the precision, recall, and F1 score of each peak calling tool. These metrics were calculated by comparing identified peaks against a reference set of high-confidence peaks, derived as described in the Methods section. Precision was defined as the proportion of peaks called by each method that overlap with the consensus set, while recall was defined as the fraction of consensus peaks successfully identified by each method. The F1 score, being the harmonic mean of precision and recall, provided a balanced measure that considered both false positives and false negatives.

**Figure 6.**
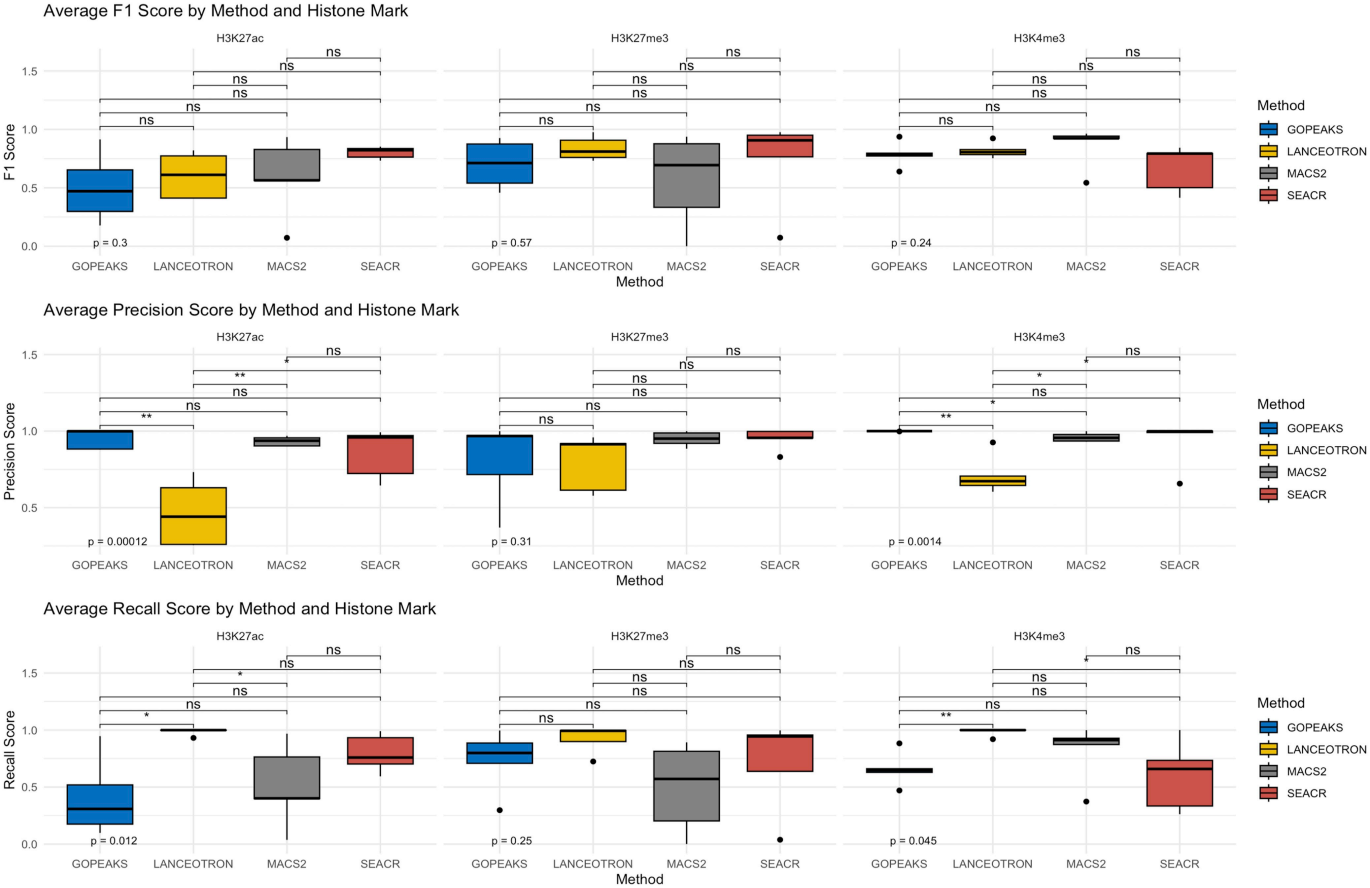
Precision, recall and F1 score for all four peak calling methods based on the histone mark tested.

Figure 6 shows the performance of each peak calling method in terms of precision across three different histone marks (H3K4me3, H3K27ac, and H3K27me3). Among the methods, GoPeaks exhibited the highest precision for H3K4me3 and H3K27ac, while SEACR performed comparably for H3K27me3. Conversely, LanceOtron displayed the lowest precision across most histone marks, which may indicate its higher rate of false positives, particularly for H3K4me3 and H3K27ac.

In terms of recall, LanceOtron and SEACR exhibited superior performance compared to MACS2 and GoPeaks for all three histone marks, with LanceOtron achieving the highest recall values for H3K27me3. This trend was consistent for LanceOtron suggesting the method to be more sensitive in detecting true positives, albeit at the potential expense of a higher number of false positives, as suggested by its relatively lower precision values.

The F1 scores illustrated in Fig. 6 for each peak caller is summarizing the trade-off between precision and recall. SEACR had the highest F1 scores for H3K27ac and H3K27me3, highlighting its balanced performance in identifying true peaks with reasonable precision and recall. MACS2 performed best for H3K4me3, while GoPeaks and LanceOtron showed comparable F1 scores.

**Figure 7.**
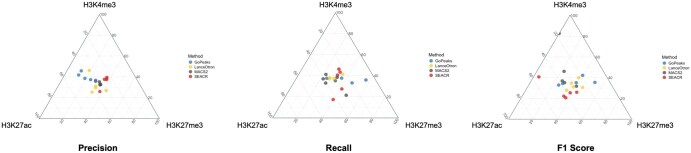
Normalized precision, recall, and F1 scores shown using ternary plots across histone marks for each peak caller, enabling visualization of trade-offs between performance metrics for each method.

Ternary plots presented in Fig. 7 illustrate the relative balance between precision, recall, and F1 score across histone marks for each peak calling method. For instance, GoPeaks tends to exhibit higher precision which makes it a suitable choice for active marks like H3K4me3, while its higher sensitivity makes it one of the top choices for broader peaks such as those found in H3K27me3. On the other hand, MACS2 and SEACR’s balanced performance in terms of precision and recall could make them suitable for any given histone mark regardless of their peak length. These metrics provide a comprehensive understanding of each method’s ability to accurately identify regions of interest, balancing sensitivity and specificity.

### 3.7 Overlap analysis between replicates

**Figure 8.**
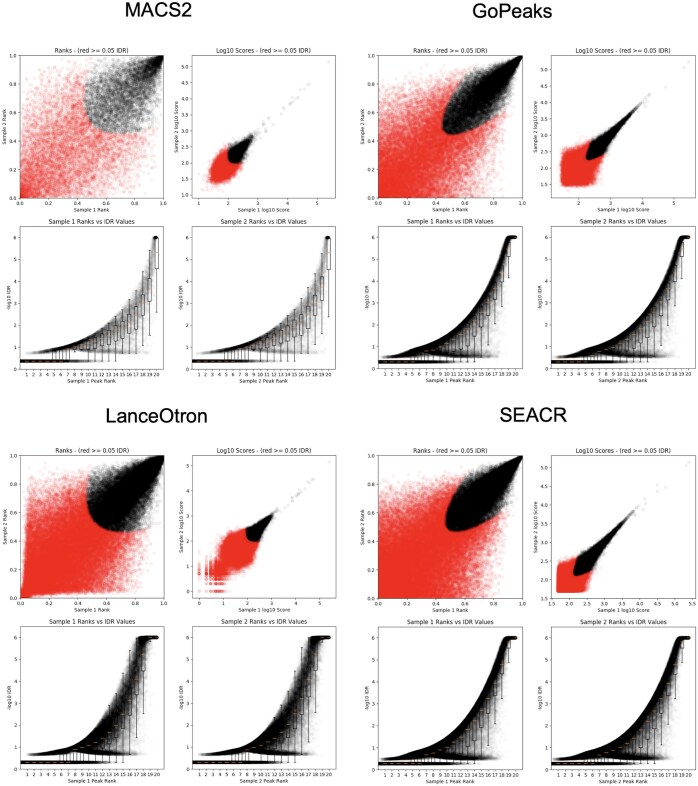
IDR analysis for the overlap between the replicates using all the four peak calling methods.

To assess reproducibility between biological replicates, we performed IDR analysis on the mouse brain CUT&RUN samples for all four peak calling methods. The IDR results, presented in Fig. 8, quantify the proportion of peaks retained at an IDR threshold of 5%, providing a direct metric for cross-replicate concordance. GoPeaks demonstrated the highest reproducibility, with 71% ± 2.3% of peaks retained across replicates, indicating robust and conservative peak detection. SEACR followed with 58% ± 3.1%, particularly effective for broader histone marks like H3K27me3. MACS2 exhibited 47% ± 2.7% reproducibility, reflecting a balance between sensitivity and reproducibility. LanceOtron, while identifying the largest number of peaks, showed lower cross-replicate agreement, with 35% ± 3.6% of peaks reproducible, suggesting potential over-sensitivity and inclusion of sample-specific peaks. These IDR-derived reproducibility scores underscore the trade-off between the sensitivity of a peak caller and its consistency across biological replicates. While LanceOtron and SEACR are more permissive in peak identification, GoPeaks maintains higher reproducibility, making it a reliable choice for studies prioritizing cross-sample consistency.

## 4 Discussion

This study provides a comprehensive benchmarking of four peak calling methods, MACS2, SEACR, GoPeaks, and LanceOtron for the analysis of CUT&RUN datasets, focusing on the histone modifications H3K4me3, H3K27ac, and H3K27me3. Our results highlight significant variability in the performance of these tools, emphasizing the importance of selecting an appropriate peak caller for the unique characteristics of CUT&RUN data. The diversity of performance metrics, including peak number, peak length distribution, SNR, and overlap across replicates, underscores the complexity of accurately identifying regions of interest and the need for tailored peak calling strategies.

The key finding of our analysis is the distinct behavior of each peak caller, reflecting differences in their sensitivity, precision, and overall efficacy. LanceOtron exhibited the highest sensitivity across all histone marks, consistently calling the largest number of peaks. However, its lower precision suggests that a substantial proportion of these peaks might represent false positives. This trade-off between sensitivity and specificity is a fundamental consideration when choosing a peak caller for a given experimental goal. For exploratory studies requiring maximal peak discovery, LanceOtron may be advantageous, while for high-confidence peak detection, additional filtering or consensus approaches may be necessary.

In contrast, GoPeaks displayed a more stringent peak calling approach, identifying fewer peaks but achieving higher precision, particularly for active histone marks such as H3K4me3 and H3K27ac. This indicates that GoPeaks is more conservative in identifying true positives, making it suitable for studies prioritizing specificity over sensitivity. However, the lower recall observed for GoPeaks implies that biologically relevant peaks with weaker signals might be missed, especially for broader repressive marks like H3K27me3.

SEACR and MACS2 showed intermediate behaviors, with SEACR demonstrating balanced performance in terms of precision and recall, particularly for H3K27ac and H3K27me3. This makes SEACR an attractive option for CUT&RUN data, where both sensitivity and precision are crucial. MACS2, a tool initially developed for ChIP-seq, also performed well for H3K4me3, suggesting its applicability in CUT&RUN despite its original design for a different type of data.

Our overlap analysis between peak callers and biological replicates further emphasizes the variability inherent in peak detection across different methods.

To facilitate standardized comparisons across methods, we constructed a coverage-based consensus peak set. Although the coverage-based consensus peak set provides a high-confidence reference for systematic benchmarking, it should be interpreted as an approximation of the ground truth rather than a definitive set and may carry inherent limitations. Conservative peak callers that identify fewer, high-confidence peaks may disproportionately influence the consensus set, inflating precision estimates for such methods while penalizing more permissive callers, which may capture biologically relevant peaks missed by conservative tools. Moreover, the method treats all overlapping regions equally, irrespective of peak shape or signal strength reported by individual methods. Additionally, merging adjacent consensus regions can obscure fine-scale resolution of binding events, particularly in complex or repetitive genomic regions where peak boundaries are ambiguous. Finally, the threshold of requiring support from three out of four callers may exclude true positives identified by only two methods, especially for challenging histone marks with diffuse signal patterns. Future improvements could integrate orthogonal experimental validation and probabilistic scoring schemes to enhance the robustness of the reference set.

While we show relatively comparable peak counts among peak callers for mouse brain samples, more pronounced discrepancies were observed in other datasets. These differences strongly correlate with dataset-specific quality metrics, such as library complexity and SNR.

These findings highlight that the apparent performance of peak callers is not solely method-dependent but also influenced by the underlying quality of the input data. Therefore, we caution against overgeneralizing global conclusions across experiments with heterogeneous data quality. When selecting an appropriate peak caller, it is important to consider sample-level metrics such as fragment size distribution, duplication rates, and background signal as potential confounding factors.

A central element of our benchmarking approach was the generation of a consensus peak set, used as a reference ground truth. While this method provides a high-confidence subset of peaks by requiring support from at least three of the four peak callers, it has some limitations which can lead to inflated precision metrics for conservative tools and higher recall for permissive ones. We acknowledge this limitation and caution against interpreting the consensus set as a definitive ground truth. Rather, it serves as a practical approximation to enable standardized comparisons.

The SNR analysis and peak length distribution also revealed method-specific biases. GoPeaks consistently identified broader peaks, particularly for H3K27me3, which may be advantageous when analyzing repressive chromatin domains that exhibit diffuse signal patterns. On the other hand, LanceOtron’s narrower peaks might be more suitable for defining sharp boundaries of active chromatin regions. These differences suggest that the optimal peak caller may vary depending on the specific histone mark and biological context under investigation.

Moreover, other works has shown that semi-supervised learning approaches can substantially improve peak calling performance in ChIP-seq by leveraging expert visual labels and machine learning to guide parameter tuning (Hocking *et al.* 2017). A similar strategy could be adapted to CUT&RUN by integrating expert-curated peak sets or orthogonal datasets as training references. Supervised models could be trained to identify optimal thresholds or scoring criteria for specific histone marks, thereby improving both sensitivity and precision. Alternatively, unsupervised models leveraging replicate concordance could be explored to optimize peak detection without the need for labeled training data (Vu *et al.* 2023). These machine learning-driven strategies represent promising directions for next-generation peak callers.

In summary, our benchmarking analysis provides answers the key questions outlined in the Introduction. We demonstrate that no single peak caller is universally optimal for CUT&RUN data. Instead, each tool presents distinct trade-offs between sensitivity, specificity, and reproducibility, which vary depending on the histone mark and biological context.

SEACR emerged as a well-balanced tool with strong performance across most metrics, particularly for repressive marks. MACS2 performed well for sharp peaks such as H3K4me3, while GoPeaks offered high precision with a conservative calling strategy. LanceOtron delivered high recall and sensitivity but at the cost of lower precision.

Reproducibility between replicates was highest for GoPeaks and SEACR, as shown by IDR analysis, whereas LanceOtron was more susceptible to variations in dataset quality.

Our results suggest that optimal peak calling may benefit from either careful parameter tuning or ensemble-based approaches that leverage the complementary strengths of multiple tools. We recommend a multi-metric evaluation framework when selecting or benchmarking tools for CUT&RUN analysis to ensure robust and reproducible results.

## Supplementary Material

btaf375_Supplementary_Data

## Data Availability

The CUT&RUN data generated in this study have been deposited in the GEO under the accession number GSE282809. This dataset includes processed peak files in BED format and genome-wide signal tracks in bigWig format for visualization. Specific datasets for H3K4me3, H3K27ac, and H3K27me3 across two biological replicates are available as part of this submission. The data will be made publicly available upon publication. All the 4D Nucleome datasets can be obtained from the 4D Nucleome Data Portal (https://data.4dnucleome.org/). The accession numbers for all 4D Nucleome datasets used are provided in Table S5, available as supplementary data at *Bioinformatics* online. All scripts used for data processing, figure generation, and analysis are available in the following GitHub repository: https://github.com/OroujiLab/CUTandRun_Peak_Calling/, and have also been archived on Zenodo (https://doi.org/10.5281/zenodo.15208150).
