# Supplementary material for: Benchmarking peak calling methods for CUT&RUN
Source: Bioinformatics. 2025 Jun 26;41(7):btaf375. doi: 10.1093/bioinformatics/btaf375 (PMC12255880; doi:10.1093/bioinformatics/btaf375)
Supplement: btaf375_Supplementary_Data [file btaf375_supplementary_data.zip › Supplementary_Data.docx]

**Supplementary Figure 1.**

**
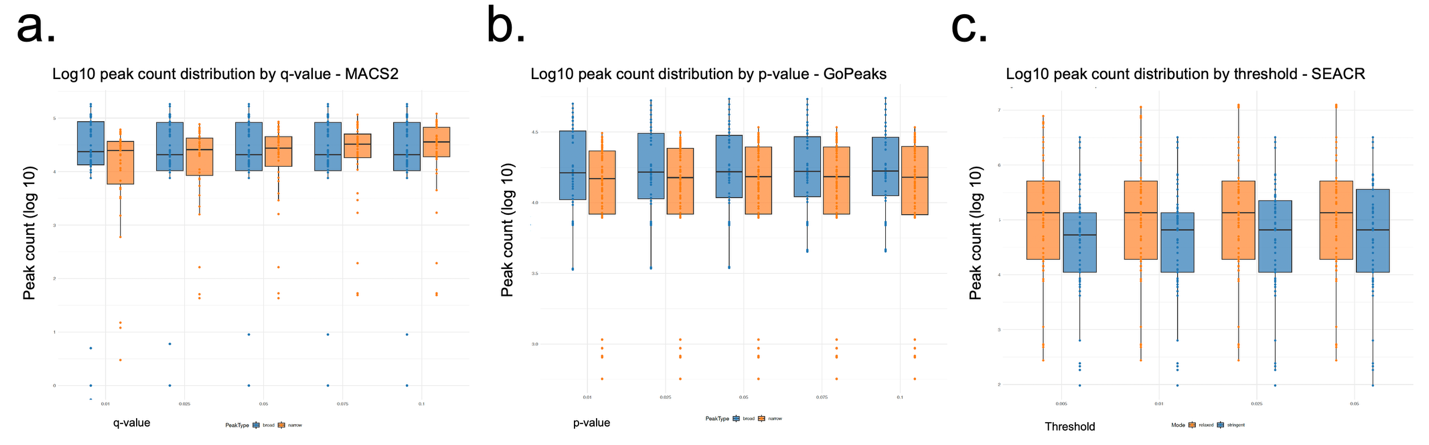
**

**Supplementary Table 1.** Sample IDs, number of replicates, origin, species, and source of data.

| Sample Name | Replicate | Origin | Species | Source |
| --- | --- | --- | --- | --- |
| GM12878_R1 | R1 | Lymphoblast | Human | 4DNucleome |
| GM12878_R2 | R2 | Lymphoblast | Human | 4DNucleome |
| H1_ESC_R1 | R1 | Embryonic Stem Cells (ESC) | Human | 4DNucleome |
| H1_ESC_R2 | R2 | Embryonic Stem Cells (ESC) | Human | 4DNucleome |
| H1_ESC_R3 | R3 | Embryonic Stem Cells (ESC) | Human | 4DNucleome |
| H1_ESC_R4 | R4 | Embryonic Stem Cells (ESC) | Human | 4DNucleome |
| H1_ESC_R5 | R5 | Embryonic Stem Cells (ESC) | Human | 4DNucleome |
| HFFc6_R1 | R1 | Foreskin Fibroblast (HFFc6) | Human | 4DNucleome |
| HFFc6_R2 | R2 | Foreskin Fibroblast (HFFc6) | Human | 4DNucleome |
| HFFc6_R3 | R3 | Foreskin Fibroblast (HFFc6) | Human | 4DNucleome |
| HFFc6_R4 | R4 | Foreskin Fibroblast (HFFc6) | Human | 4DNucleome |
| K562_R1 | R1 | Myelogenous Leukemia | Human | 4DNucleome |
| K562_R2 | R2 | Myelogenous Leukemia | Human | 4DNucleome |
| Mouse_Brain_R1 | R1 | Brain | Mouse | In-house* |
| Mouse_Brain_R2 | R2 | Brain | Mouse | In-house* |

* Mouse brain samples were generated in-house (Princess Margaret Cancer Centre); all other datasets were obtained from the 4DNucleome portal.

**Supplementary Table 2.** IgG enrichment ratios for all CUT&RUN samples.

| **Sample_Name** | **Total_Reads** | **mark** | **cell_line** | **replicate** | **IgG_Reads** | **ratio** |
| --- | --- | --- | --- | --- | --- | --- |
| H3K4me3_GM12878_R1 | 20795986 | H3K4me3 | GM12878 | 1 | 10242218 | 2.030418216 |
| H3K4me3_GM12878_R2 | 27399004 | H3K4me3 | GM12878 | 2 | 24824424 | 1.103711571 |
| H3K4me3_H1_ESC_R1 | 26586760 | H3K4me3 | H1 | 1 | 19138296 | 1.389191598 |
| H3K4me3_H1_ESC_R2 | 17970828 | H3K4me3 | H1 | 2 | 11308512 | 1.589141701 |
| H3K4me3_H1_ESC_R3 | 13588216 | H3K4me3 | H1 | 3 | 13562530 | 1.001893894 |
| H3K4me3_H1_ESC_R4 | 29095414 | H3K4me3 | H1 | 4 | 15791111 | 1.84251849 |
| H3K4me3_H1_ESC_R5 | 30890106 | H3K4me3 | H1 | 5 | 14364722 | 2.150414467 |
| H3K4me3_HFFc6_R1 | 20663348 | H3K4me3 | HFFc6 | 1 | 3498504 | 5.906338252 |
| H3K4me3_HFFc6_R2 | 17355700 | H3K4me3 | HFFc6 | 2 | 10756686 | 1.613480211 |
| H3K4me3_HFFc6_R3 | 22381762 | H3K4me3 | HFFc6 | 3 | 12728949 | 1.758335429 |
| H3K4me3_HFFc6_R4 | 17984442 | H3K4me3 | HFFc6 | 4 | NA | NA |
| H3K4me3_K562_R1 | 17198924 | H3K4me3 | K562 | 1 | 22534074 | 0.76324077 |
| H3K4me3_K562_R2 | 26870120 | H3K4me3 | K562 | 2 | 19392338 | 1.385604975 |
| H3K4me3_Mouse_Brain_R1 | 104530042 | H3K4me3 | Mouse | 1 | NA | NA |
| H3K4me3_Mouse_Brain_R2 | 83002416 | H3K4me3 | Mouse | 2 | NA | NA |
| H3K27ac_GM12878_R1 | 14997878 | H3K27ac | GM12878 | 1 | 10242218 | 1.46431935 |
| H3K27ac_GM12878_R2 | 18988768 | H3K27ac | GM12878 | 2 | 24824424 | 0.764922803 |
| H3K27ac_H1_ESC_R1 | 22194238 | H3K27ac | H1 | 1 | 19138296 | 1.159676807 |
| H3K27ac_H1_ESC_R2 | 21570064 | H3K27ac | H1 | 2 | 11308512 | 1.907418412 |
| H3K27ac_H1_ESC_R3 | 33455864 | H3K27ac | H1 | 3 | 13562530 | 2.466786359 |
| H3K27ac_H1_ESC_R4 | 27021164 | H3K27ac | H1 | 4 | 15791111 | 1.711162945 |
| H3K27ac_H1_ESC_R5 | 27980952 | H3K27ac | H1 | 5 | 14364722 | 1.947893736 |
| H3K27ac_K562_R1 | 24102180 | H3K27ac | K562 | 1 | 22534074 | 1.069588216 |
| H3K27ac_K562_R2 | 14903622 | H3K27ac | K562 | 2 | 19392338 | 0.768531468 |
| H3K27ac_HFFc6_R1 | 20265536 | H3K27ac | HFFc6 | 1 | 3498504 | 5.792629078 |
| H3K27ac_HFFc6_R2 | 18117348 | H3K27ac | HFFc6 | 2 | 10756686 | 1.684287149 |
| H3K27ac_HFFc6_R3 | 15146690 | H3K27ac | HFFc6 | 3 | 12728949 | 1.189940348 |
| H3K27ac_HFFc6_R4 | 17354642 | H3K27ac | HFFc6 | 4 | NA | NA |
| H3K27ac_Mouse_Brain_R1 | 76972944 | H3K27ac | Mouse | 1 | NA | NA |
| H3K27ac_Mouse_Brain_R2 | 75386724 | H3K27ac | Mouse | 2 | NA | NA |
| H3K27me3_GM12878_R1 | 18205060 | H3K27me3 | GM12878 | 1 | 10242218 | 1.777452892 |
| H3K27me3_GM12878_R2 | 42031282 | H3K27me3 | GM12878 | 2 | 24824424 | 1.693142286 |
| H3K27me3_H1_ESC_R1 | 21482234 | H3K27me3 | H1 | 1 | 19138296 | 1.122473704 |
| H3K27me3_H1_ESC_R2 | 16064858 | H3K27me3 | H1 | 2 | 11308512 | 1.420598749 |
| H3K27me3_H1_ESC_R3 | 16291224 | H3K27me3 | H1 | 3 | 13562530 | 1.201193583 |
| H3K27me3_H1_ESC_R4 | 31149788 | H3K27me3 | H1 | 4 | 15791111 | 1.972615353 |
| H3K27me3_H1_ESC_R5 | 25745476 | H3K27me3 | H1 | 5 | 14364722 | 1.792271093 |
| H3K27me3_HFFc6_R1 | 20993134 | H3K27me3 | HFFc6 | 1 | 3498504 | 6.000603115 |
| H3K27me3_HFFc6_R2 | 14067486 | H3K27me3 | HFFc6 | 2 | 10756686 | 1.307789964 |
| H3K27me3_HFFc6_R3 | 23328196 | H3K27me3 | HFFc6 | 3 | 12728949 | 1.832688308 |
| H3K27me3_HFFc6_R4 | 20227100 | H3K27me3 | HFFc6 | 4 | NA | NA |
| H3K27me3_K562_R1 | 17843370 | H3K27me3 | K562 | 1 | 22534074 | 0.791839505 |
| H3K27me3_K562_R2 | 25429284 | H3K27me3 | K562 | 2 | 19392338 | 1.311305733 |
| H3K27me3_Mouse_Brain_R1 | 75718796 | H3K27me3 | Mouse | 1 | NA | NA |
| H3K27me3_Mouse_Brain_R2 | 88012518 | H3K27me3 | Mouse | 2 | NA | NA |

**Supplementary Table 3.** Total number of reads per CUT&RUN library.

| **Sample_Name** | **Total_Reads** |
| --- | --- |
| 4DNFIQ69E2QT_H3K4me3_GM12878_R1 | 20795986 |
| 4DNFIP9JYOC7_H3K4me3_GM12878_R2 | 27399004 |
| 4DNFIBC19N9Z_H3K4me3_H1_ESC_R1 | 26586760 |
| 4DNFIU2WN75X_H3K4me3_H1_ESC_R2 | 17970828 |
| 4DNFIFLCCOGI_H3K4me3_H1_ESC_R3 | 13588216 |
| 4DNFIQKTWL8V_H3K4me3_H1_ESC_R4 | 29095414 |
| 4DNFIR98Z897_H3K4me3_H1_ESC_R5 | 30890106 |
| 4DNFIQR8QYNC_H3K4me3_HFFc6_R1 | 20663348 |
| 4DNFIKL6UY2S_H3K4me3_HFFc6_R2 | 17355700 |
| 4DNFIXY51HWA_H3K4me3_HFFc6_R3 | 22381762 |
| 4DNFIB86FQ1K_H3K4me3_HFFc6_R4 | 17984442 |
| 4DNFIA9FCYJ6_H3K4me3_K562_R1 | 17198924 |
| 4DNFI4WI95J5_H3K4me3_K562_R2 | 26870120 |
| mm10_H3K4me3_Mouse_Brain_R1 | 104530042 |
| mm10_H3K4me3_Mouse_Brain_R2 | 83002416 |
|  |  |
| 4DNFIU38AOAH_H3K27ac_GM12878_R1 | 14997878 |
| 4DNFIPCWBPL6_H3K27ac_GM12878_R2 | 18988768 |
| 4DNFILVB7TTJ_H3K27ac_H1_ESC_R1 | 22194238 |
| 4DNFIFJNJRAL_H3K27ac_H1_ESC_R2 | 21570064 |
| 4DNFI5ONYZ4L_H3K27ac_H1_ESC_R3 | 33455864 |
| 4DNFIJMSH53P_H3K27ac_H1_ESC_R4 | 27021164 |
| 4DNFIT2FWPBP_H3K27ac_H1_ESC_R5 | 27980952 |
| 4DNFIPFWFCL6_H3K27ac_K562_R1 | 24102180 |
| 4DNFINP6XLH9_H3K27ac_K562_R2 | 14903622 |
| 4DNFITMH1HEV_H3K27ac_HFFc6_R1 | 20265536 |
| 4DNFIM1AAQLD_H3K27ac_HFFc6_R2 | 18117348 |
| 4DNFIJR7PZ69_H3K27ac_HFFc6_R3 | 15146690 |
| 4DNFI69M1D4T_H3K27ac_HFFc6_R4 | 17354642 |
| mm10_H3K27ac_Mouse_Brain_R1 | 76972944 |
| mm10_H3K27ac_Mouse_Brain_R2 | 75386724 |
|  |  |
| 4DNFI74K8QSV_H3K27me3_GM12878_R1 | 18205060 |
| 4DNFIMJZ5B1V_H3K27me3_GM12878_R2 | 42031282 |
| 4DNFIE1XUS5M_H3K27me3_H1_ESC_R1 | 21482234 |
| 4DNFIVEYA5ZH_H3K27me3_H1_ESC_R2 | 16064858 |
| 4DNFILOOTLJA_H3K27me3_H1_ESC_R3 | 16291224 |
| 4DNFIX1MXHWY_H3K27me3_H1_ESC_R4 | 31149788 |
| 4DNFIT4JBYK2_H3K27me3_H1_ESC_R5 | 25745476 |
| 4DNFIRNNW2QD_H3K27me3_HFFc6_R1 | 20993134 |
| 4DNFI59RK6U6_H3K27me3_HFFc6_R2 | 14067486 |
| 4DNFIZEABOGS_H3K27me3_HFFc6_R3 | 23328196 |
| 4DNFI5BWH5R8_H3K27me3_HFFc6_R4 | 20227100 |
| 4DNFIBDJW6IC_H3K27me3_K562_R1 | 17843370 |
| 4DNFIRWKCRVO_H3K27me3_K562_R2 | 25429284 |
| mm10_H3K27me3_Mouse_Brain_R1 | 75718796 |
| mm10_H3K27me3_Mouse_Brain_R2 | 88012518 |

**Supplementary Table 4.** Number of overlapping peaks among four or three peak-calling methods across various samples and histone marks.

| Sample | Overlap Type | H3K27ac | H3K4me3 | H3K27me3 |
| --- | --- | --- | --- | --- |
| H1_ESC | 4-Methods | 24,501 | 23,502 | 312 |
|  | 3-Methods [4 - SEACR] | 9 | 722 | 10,393 |
|  | 3-Methods [4 - MACS2] | 1,955 | 19 | 0 |
|  | 3-Methods [4 - LanceOtron] | 0 | 0 | 0 |
|  | 3-Methods [4 - GoPeaks] | 61,707 | 9,444 | 0 |
| K562 | 4-Methods | 18,099 | 6,568 | 16,098 |
|  | 3-Methods [4 - SEACR] | 60 | 9,586 | 3,051 |
|  | 3-Methods [4 - MACS2] | 21 | 0 | 241 |
|  | 3-Methods [4 - LanceOtron] | 0 | 0 | 198 |
|  | 3-Methods [4 - GoPeaks] | 40,482 | 0 | 827 |
| HFFc6 | 4-Methods | 4,091 | 17,717 | 9 |
|  | 3-Methods [4 - SEACR] | 68 | 421 | 0 |
|  | 3-Methods [4 - MACS2] | 26,703 | 12,982 | 18,704 |
|  | 3-Methods [4 - LanceOtron] | 0 | 0 | 0 |
|  | 3-Methods [4 - GoPeaks] | 26 | 0 | 0 |
| GM12878 | 4-Methods | 16,691 | 6,982 | 20,054 |
|  | 3-Methods [4 - SEACR] | 1,142 | 7,618 | 6,054 |
|  | 3-Methods [4 - MACS2] | 23 | 0 | 3,627 |
|  | 3-Methods [4 - LanceOtron] | 0 | 0 | 2 |
|  | 3-Methods [4 - GoPeaks] | 11,690 | 0 | 5,412 |
| Mouse_Brain | 4-Methods | 26,824 | 43,378 | 8,638 |
|  | 3-Methods [4 - SEACR] | 408 | 1 | 95 |
|  | 3-Methods [4 - MACS2] | 32,331 | 2,510 | 2,548 |
|  | 3-Methods [4 - LanceOtron] | 1,437 | 1,863 | 1,965 |
|  | 3-Methods [4 - GoPeaks] | 602 | 1,584 | 76 |

**Supplementary Table 5.** Accession numbers for 4D Nucleome datasets used in this study.

| Accession Number | Dataset | Replicate Info |
| --- | --- | --- |
| 4DNFIU38AOAH | H3K27ac___GM12878 | Biorep_1 |
| 4DNFIPCWBPL6 | H3K27ac___GM12878 | Biorep_2 |
| 4DNFI258RO3L | H3K27ac___H1_ESC | Biorep_1 |
| 4DNFIYET6YFK | H3K27ac___H1_ESC | Biorep_2 |
| 4DNFI5ONYZ4L | H3K27ac___H1_ESC | Biorep_3 |
| 4DNFIJMSH53P | H3K27ac___H1_ESC | Biorep_4 |
| 4DNFIT2FWPBP | H3K27ac___H1_ESC | Biorep_5 |
| 4DNFITMH1HEV | H3K27ac___HFFc6 | Biorep_1 |
| 4DNFIM1AAQLD | H3K27ac___HFFc6 | Biorep_2 |
| 4DNFIJR7PZ69 | H3K27ac___HFFc6 | Biorep_3 |
| 4DNFI69M1D4T | H3K27ac___HFFc6 | Biorep_4 |
| 4DNFIPFWFCL6 | H3K27ac___K562 | Biorep_1 |
| 4DNFINP6XLH9 | H3K27ac___K562 | Biorep_2 |
| 4DNFI74K8QSV | H3K27me3___GM12878 | Biorep_1 |
| 4DNFIMJZ5B1V | H3K27me3___GM12878 | Biorep_2 |
| 4DNFIE1XUS5M | H3K27me3___H1_ESC | Biorep_1 |
| 4DNFIVEYA5ZH | H3K27me3___H1_ESC | Biorep_2 |
| 4DNFILOOTLJA | H3K27me3___H1_ESC | Biorep_3 |
| 4DNFIX1MXHWY | H3K27me3___H1_ESC | Biorep_4 |
| 4DNFIT4JBYK2 | H3K27me3___H1_ESC | Biorep_5 |
| 4DNFIRNNW2QD | H3K27me3___HFFc6 | Biorep_1 |
| 4DNFI59RK6U6 | H3K27me3___HFFc6 | Biorep_2 |
| 4DNFIZEABOGS | H3K27me3___HFFc6 | Biorep_3 |
| 4DNFI5BWH5R8 | H3K27me3___HFFc6 | Biorep_4 |
| 4DNFIBDJW6IC | H3K27me3___K562 | Biorep_1 |
| 4DNFIRWKCRVO | H3K27me3___K562 | Biorep_2 |
| 4DNFIQ69E2QT | H3K4me3___GM12878 | Biorep_1 |
| 4DNFIP9JYOC7 | H3K4me3___GM12878 | Biorep_2 |
| 4DNFIBC19N9Z | H3K4me3___H1_ESC | Biorep_1 |
| 4DNFIU2WN75X | H3K4me3___H1_ESC | Biorep_2 |
| 4DNFIFLCCOGI | H3K4me3___H1_ESC | Biorep_3 |
| 4DNFIQKTWL8V | H3K4me3___H1_ESC | Biorep_4 |
| 4DNFIR98Z897 | H3K4me3___H1_ESC | Biorep_5 |
| 4DNFIQR8QYNC | H3K4me3___HFFc6 | Biorep_1 |
| 4DNFIKL6UY2S | H3K4me3___HFFc6 | Biorep_2 |
| 4DNFIXY51HWA | H3K4me3___HFFc6 | Biorep_3 |
| 4DNFIB86FQ1K | H3K4me3___HFFc6 | Biorep_4 |
| 4DNFIA9FCYJ6 | H3K4me3___K562 | Biorep_1 |
| 4DNFI4WI95J5 | H3K4me3___K562 | Biorep_2 |
| 4DNFI1PXR4LE | IgG_control___GM12878 | Biorep_1 |
| 4DNFIW3M5OJ6 | IgG_control___GM12878 | Biorep_2 |
| 4DNFI8VSO2EO | IgG_control___H1_ESC | Biorep_1 |
| 4DNFIY4OI3IW | IgG_control___H1_ESC | Biorep_2 |
| 4DNFICFGSHE1 | IgG_control___H1_ESC | Biorep_3 |
| 4DNFI2167UID | IgG_control___H1_ESC | Biorep_4 |
| 4DNFIPRQIZAP | IgG_control___H1_ESC | Biorep_5 |
| 4DNFIJ7MBX1U | IgG_control___HFFc6 | Biorep_1 |
| 4DNFIL9L5484 | IgG_control___HFFc6 | Biorep_2 |
| 4DNFI1VPYOON | IgG_control___HFFc6 | Biorep_3 |
| 4DNFIWTC4SAI | IgG_control___K562 | Biorep_1 |
| 4DNFIL1CWQJR | IgG_control___K562 | Biorep_2 |
