# Supplementary figures and images for: Benchmarking peak calling methods for CUT&RUN
Source: Bioinformatics. 2025 Jun 26;41(7):btaf375. doi: 10.1093/bioinformatics/btaf375 (PMC12255880; doi:10.1093/bioinformatics/btaf375)

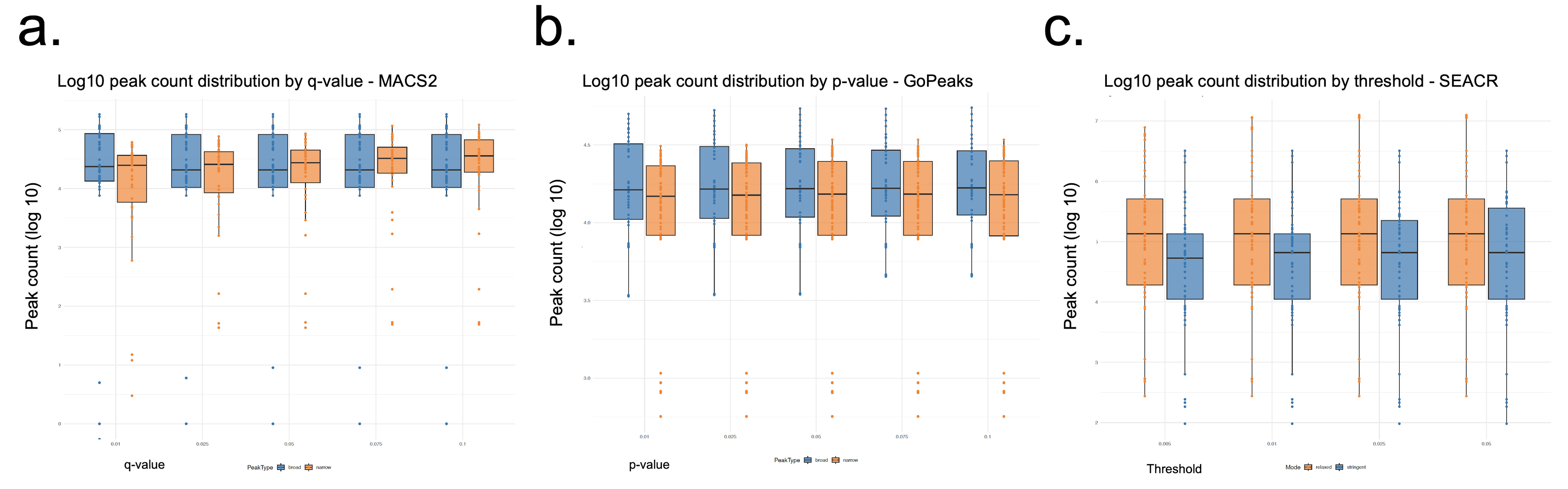

Supplement: btaf375_Supplementary_Data [file btaf375_supplementary_data.zip › Supp_Figure1.png]
